# A Survey of Deep Learning-Based 3D Object Detection Methods for Autonomous Driving Across Different Sensor Modalities

**DOI:** 10.3390/s25175264

**Published:** 2025-08-24

**Authors:** Miguel Valverde, Alexandra Moutinho, João-Vitor Zacchi

**Affiliations:** 1 Instituto Superior Técnico, Universidade de Lisboa, 1049-001 Lisbon, Portugal; 2 Instituto de Engenharia Mecânica, Instituto Superior Técnico, Universidade de Lisboa, 1049-001 Lisbon, Portugal; 3 Fraunhofer IKS, 80686 Munich, Germany; joao-vitor.zacchi@iks.fraunhofer.de

**Keywords:** 3D object detection, deep learning, monocular camera, stereo vision, LiDAR, radar, sensor fusion, KITTI, nuScenes, Waymo, autonomous vehicles

## Abstract

This paper presents a comprehensive survey of deep learning-based methods for 3D object detection in autonomous driving, focusing on their use of diverse sensor modalities, including monocular cameras, stereo vision, LiDAR, radar, and multi-modal fusion. To systematically organize the literature, a structured taxonomy is proposed that categorizes methods by input modality. The review also outlines the chronological evolution of these approaches, highlighting major architectural developments and paradigm shifts. Furthermore, the surveyed methods are quantitatively compared using standard evaluation metrics across benchmark datasets in autonomous driving scenarios. Overall, this work provides a detailed and modality-agnostic overview of the current landscape of deep learning approaches for 3D object detection in autonomous driving. Results of this work are available in a github open repository.

## 1. Introduction

### 1.1. Overview of Autonomous Vehicles

Autonomous vehicles (AVs) have emerged as a transformative paradigm in mobility, with the potential to reshape how people and goods are transported and how societies function. Over the past decade, significant advances in sensor technology, computational power, and machine learning have enabled the transition from early concepts to real-world deployment. AVs are now being tested and operated in urban areas, industrial settings, and controlled environments worldwide. Governments and companies continue to invest heavily in their development, driven by the promise of reducing traffic accidents, lowering emissions, and improving effectiveness and efficiency in the transportation of people.

**Figure 1:**
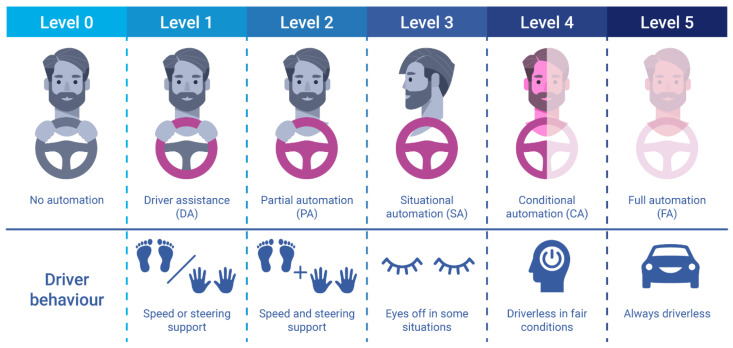
SAE levels of automation [1].

According to the Society of Automotive Engineers’ (SAE) norm for driving automation [1], there are six distinct levels (illustrated in Figure 1), ranging from level 0, where the driver is in command of the car, to level 5, where the vehicle assumes complete control over all driving aspects. Levels 1 and 2 of automation, which include advanced driver assistance systems (ADASs) such as braking assistance, cruise control, and lane switching [2], have now practically become standard in many cars. Safety concerns primarily drove their adoption but also paved the way for the development of more advanced systems.

For a long time, the automotive industry was stuck at SAE Level 2; however, vehicles with Level 3 automation have recently started entering the market. Notably, the Mercedes S-Class achieved Level 3 certification in Germany and in the United States, while the Honda Legend received the same certification in Japan [3]. Moreover, robotaxis are already operating at Level 4 [4], though some argue they can be considered Level 5 due to their full autonomy in constrained environments [5].

Safety is a key driver of AD growth. Studies such as [6,7] highlight that approximately 90% to 94% of accidents are caused by human error. AVs aim to mitigate these risks by eliminating unsafe driver behaviours like distraction and speeding. Additionally, AVs are expected to reduce carbon emissions, smooth traffic flow, increase productivity, and have a positive economic impact. A study from America’s Workforce and Self-Driving Future [8] predicts that widespread adoption of AVs could lead to nearly $800 billion in annual social and economic benefits by 2050. Another study [9] states that autonomous technologies can reduce urban travel time by a third, decrease greenhouse gas emissions by two-thirds, decrease the number of vehicles in crowded cities by 30%, and decrease the need for parking spaces by 40%.

**Figure 2:**
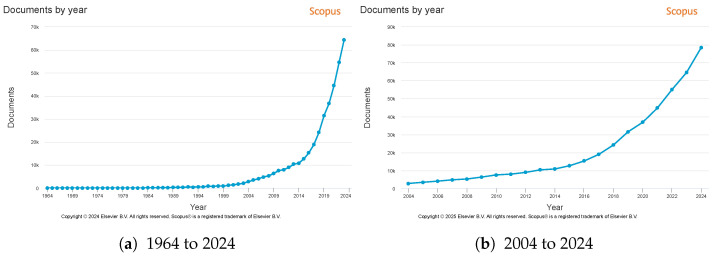
Number of publications per year on autonomous vehicles. Source Scopus—Elsevier (https://www.scopus.com/home.uri— advanced search using (self-driving car) OR (autonomous car) keywords on 25 April 2025).

Autonomous driving (AD), computer vision (CV), robotics, and machine learning (ML) are among the most prominent areas of research at the moment [10]. AVs are the next step in the evolution of the automobile, as the industry is noticeably moving towards a world where less or even no human interaction is required. Figure 2a illustrates the trajectory of publications in AD since 1964, demonstrating a notable surge at the beginning of the 21st century. Figure 2b zooms on the last two decades (2004 to 2024), revealing a consistent annual publication growth. Over this period, the number of scientific publications on AD published by year increased from 2828 to 78,602, marking an almost 30-fold increase.

The anticipated benefits of a global shift toward full or partial AVs are widely recognized. However, achieving large-scale deployment remains a complex challenge and is far from imminent. Despite substantial investment and rapid technological progress, fully automated vehicles still face significant obstacles across multiple domains. Concerns over AV safety remain widespread, particularly among vulnerable groups such as individuals with disabilities [11]. Moreover, documented instances of algorithmic bias, including decisions influenced by race, further complicate public trust and acceptance [12].

Not only ethical but technical challenges still persist. Ensuring reliable performance under adverse weather conditions, such as heavy rain, snow, or fog, and in difficult lighting environments, including night-time driving, tunnels, and shaded areas, remains difficult. These scenarios can impair sensor accuracy and disrupt vehicle control systems, underscoring the need for more robust algorithms and advanced sensor fusion techniques to guarantee dependable operation across varied environments [10]. Given the substantial societal and safety benefits AVs promise, it is critical to address both technical and ethical barriers to enable their successful integration into modern transportation systems.

**Figure 3:**
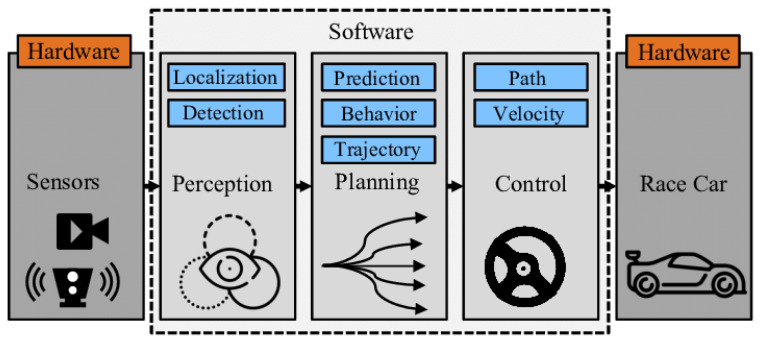
Typical autonomous vehicle pipeline [15].

An AV must perform all the functions a human driver would, including perceiving the environment, determining its position, anticipating the actions of other road users, planning a trajectory, and executing control commands to follow that trajectory. To achieve this, AV software is typically organized into distinct modules and submodules. While variations exist, a common framework follows the Sense–Plan–Act paradigm [13], illustrated in Figure 3, and includes the following components: Sensors, Perception, Planning, Control, and Car.

The Sensors module is responsible for acquiring data from a range of sensors, including cameras, Radio Detection and Ranging (radar), Light Detection and Ranging (LiDAR), Global Navigation Satellite Systems (GNSSs), and inertial sensors. The Perception module interprets these data to detect objects and estimate the state of the vehicle. Based on the output of perception, the vehicle plans a trajectory and generates control actions, such as steering and acceleration, to follow the planned path [14].

Some of the most complex challenges in autonomous driving stem from the perception stage, which relies on a combination of advanced sensors and state-of-the-art (SoA) algorithms. As an initial stage in the AV software pipeline, the performance of the Perception module directly affects all downstream tasks. Therefore, a thorough understanding of the various techniques used in perception is essential to advance reliable and safe autonomous driving systems.

### 1.2. Scope, Aims, and Outline

Three-dimensional object detection plays a central role in autonomous driving by enabling the vehicle to understand and interact with its environment in three dimensions. It involves estimating the position, dimensions, and class of objects in the environment using sensor data. Modern perception systems typically rely on multimodal inputs, such as images from cameras, point clouds from LiDAR, or a combination of both, to extract geometric and semantic information from the scene. With the rapid progress of deep learning (DL), a wide range of 3D object detection methods have emerged, each tailored to specific sensor modalities and data representations. However, these methods are often developed and evaluated in isolation, lacking a unified perspective that spans different input types and architectural paradigms. This fragmentation makes it difficult to assess their relative strengths and limitations, particularly in the context of real-world autonomous driving scenarios. To address this gap, this work presents a structured and comparative review of 3D object detection methods across all major sensor modalities, aiming to clarify performance trends, highlight paradigm shifts, and guide future developments in the field.

This work aims to provide a comprehensive review of 3D object detection methods tailored for AD, offering an in-depth analysis and structured comparison of different approaches and associated sensor modalities. Unlike existing surveys, our review includes more recent developments and evaluates methods across all types of sensory and application contexts. Although some previous works, such as [16,17,18] present similar structures, they were published before 2023 and cover older methods. More recent surveys like [19,20,21] either lack breadth or fail to benchmark and compare methods on standard datasets. Other studies focus narrowly on specific modalities, such as [22] for image-based methods, [23] for point cloud-based DL methods, and [24] for multi-modal techniques. Transformer-based approaches are explored in [25,26].

This survey presents the first unified and structured review of recent 3D object detection methods using DL across all major sensor modalities. It introduces a refined taxonomy and highlights performance trends and paradigm shifts, to support fair and systematic evaluation in future research. The survey covers developments over the past decade, focusing particularly on methods published in top-tier computer vision venues. Alongside technical insights into 3D detectors, the paper discusses taxonomies, benchmark datasets, evaluation protocols, and open challenges. More specifically, the following contributions are provided:

- The context for the task of 3D object detection, presenting its formulation, the sensor modalities required for it, and finally by presenting benchmark datasets and their respective evaluation metrics.
- A comprehensive literature review of camera-based, LiDAR-based, radar-based and multimodal-based 3D perception methods, including an updated taxonomy and discussion of their evolution.
- A performance and speed benchmark of selected 3D object detectors using standard datasets and evaluation metrics.

An updated project page listing all methods covered in this work is also maintained at (https://3d-object-detection-hub.github.io/, accessed on 25 April 2025).

The structure of this paper is organized as follows. Section 2 introduces the 3D object detection problem, followed by typical data representations and different sensor types. Then, Section 3 summarizes the commonly used datasets and metrics for 3D object detection. Section 4 presents the taxonomy of the methods considered and highlights the major paradigm shifts and developments. Finally, a speed and performance analysis is provided in Section 5. Section 6 offers concluding remarks and discusses future research trends.

## 2. Background

### 2.1. Problem Definition

Object detection (OD) is a fundamental task in CV, aiming to identify and localize objects of interest from sensory data such as images or point clouds (PCs) [27]. It typically involves a two-stage process: classifying object types and regressing bounding box parameters. While 2D OD has achieved strong performance in benchmarks like KITTI [28], its lack of depth information limits its usefulness in autonomous driving, particularly for critical tasks such as motion planning and collision avoidance [29].

Three-dimensional OD addresses these shortcomings by incorporating depth, allowing objects to be located and characterized in three-dimensional space. This additional dimensionality improves spatial reasoning but introduces greater computational demands and requires high-quality sensory inputs. Unlike its 2D counterpart, 3D OD still faces major challenges, including fewer large-scale annotated datasets and less standardized model architectures [29]. Additionally, estimating depth from monocular images remains an inherently ill-posed problem, as multiple 3D scenes can project to similar 2D views [16,30].

Formally, 3D OD aims to infer a set of bounding boxes $B=\{B_{1},B_{2},\ldots ,B_{N}\}$ from sensory input $I_{\mathrm{sensor}}$, using a detection model $f_{\det}$:

(1) $$B=f_{\det}\left(I_{\mathrm{sensor}}\right).$$

Each detected object $B_{i}$ is typically represented as:

(2) $$B_{i}=\left[x_{c},y_{c},z_{c},l,w,h,\theta ,c\right],$$

where $\left(x_{c},y_{c},z_{c}\right)$ denote the centre coordinates, (l,w,h) the bounding box dimensions length, width, and height, θ is the rotation angle around the z-axis in the world frame, and *c* represents the object category [18]. The bounding boxes are usually parametrized in sensor-centric or vehicle-centric coordinate frames to support downstream tasks such as object tracking, motion prediction, and trajectory planning [16].

### 2.2. Data Representation

Three-dimensional data provides richer spatial structure than 2D imagery and can be represented either implicitly, via depth cues embedded in images, or explicitly, through direct geometric encoding. Implicit representations include multi-view images, RGB-D formats that append depth maps to RGB channels, and light fields, which encode light rays as functions of spatial and angular dimensions [31].

Explicit representations are more common in 3D OD and include the following:

- **Voxels**, which discretize 3D space into volumetric grids;
- **Point clouds**, composed of unordered 3D points (x,y,z), sometimes augmented with intensity or reflectance values;
- **Meshes**, which represent object surfaces through vertices, edges, and faces [31].

Among these, point clouds, particularly those obtained from LiDAR, are the most widely used input for 3D OD due to their high geometric fidelity. However, their sparse and irregular structure poses challenges for conventional DL models. Voxelization addresses this by converting point clouds into regular 3D grids, enabling the use of 3D convolutions. Yet, this comes with trade-offs: small voxels increase memory and computation costs cubically, while large voxels reduce spatial resolution and accuracy [16,32].

To balance efficiency and detail, pillar-based representations simplify voxelization by collapsing the vertical dimension, effectively projecting the 3D point cloud into a pseudo-image format. This allows 2D convolutional neural networks to be used while preserving horizontal spatial structure, significantly improving computational efficiency without sacrificing detection accuracy [31].

### 2.3. Sensors

Several types of sensors can provide raw data for 3D OD. The most widely adopted sensors in AVs are radar, LiDAR, and cameras. Their integration is essential for environmental perception, directly impacting system safety and reliability.

Based on their interaction with the environment, sensors are divided into active and passive types. Active sensors, such as radar and LiDAR, emit energy signals and measure the reflected responses. Passive sensors, such as cameras, capture ambient energy without emitting any signal. Alternatively, sensors can also be categorized based on the type of information they measure:

- **Exteroceptive sensors** measure external variables and observe the surrounding environment. Examples include stereo, flash, infrared, and thermal cameras, as well as radar, LiDAR, and sonar [33].
- **Proprioceptive sensors** measure variables related to the vehicle state, providing information about its position, velocity, orientation, and acceleration. Examples include global navigation satellite systems (GNSSs), inertial measurement units (IMUs), ground speed sensors (GSSs), encoders, gyroscopes, and accelerometers [33].

While some proprioceptive information may support perception tasks, these sensors are primarily used for vehicle state estimation. In contrast, exteroceptive sensors are mainly responsible for detecting static and dynamic objects in the environment. Table 1 summarizes the main categories of exteroceptive sensors and compares them based on data from [2,33,34,35].

It is important to note that Table 1 provides only a qualitative overview. The classification depends on the specific application and the relative comparison between the considered sensors. Moreover, performance can vary significantly across different models and manufacturers. Therefore, the table should serve only as a general guideline for assessing trade-offs between different sensing modalities. In the following, these trade-offs are further examined and discussed in detail for each individual sensor modality.

**Monocular cameras** are passive sensors that capture rich appearance information, including texture and colour, at low cost and high resolution. They produce images $I_{\mathrm{cam}}\in R^{W\;\times \;H\;\times \;3}$, but cannot directly recover depth, limiting their 3D localization capabilities [2,18]. Their performance deteriorates under adverse lighting conditions such as night-time, glare, fog, or rain [29].

**Stereo vision** systems estimate depth by triangulating points based on the disparity between images captured by two horizontally aligned cameras, enhancing 3D understanding. However, they require precise calibration and are sensitive to low-texture regions and lighting variations [16,29]. Other systems, such as Time-of-Flight cameras, infer depth using infrared pulses but offer lower resolution, while RGB-D sensors like Kinect combine colour and depth for a more complete spatial view.

**Infrared cameras** detect infrared radiation, including thermal emissions and other wavelengths of the electromagnetic spectrum. They enable perception in dark or low-visibility conditions but typically provide low resolution and are less effective for detailed object classification.

**Sonar and ultrasonic** sensors emit sound waves to detect nearby obstacles. They are compact, inexpensive, and reliable at short range, but provide low spatial resolution and are unsuitable for complex 3D perception tasks [2].

**Radar** systems emit electromagnetic waves and detect their reflections to measure the position and relative velocity of objects using the Doppler effect. They provide long-range robustness and function reliably in adverse weather, although their angular resolution is lower, making fine-grained object detection more challenging [33].

**LiDAR** sensors actively scan the environment with laser beams to generate detailed 3D PCs. A typical LiDAR unit emitting *m* beams over *n* rotations produces a range image $I_{\mathrm{range}}\in R^{m\;\times \;n\;\times \;3}$, which can be converted into a PC $I_{\mathrm{point}}\in R^{N\;\times \;3}$ [18,29]. LiDAR provides high spatial accuracy independent of light conditions, although it remains relatively expensive and can be affected by environmental factors like fog, rain, or snow [16].

Each sensing modality presents unique trade-offs. Monocular cameras offer high semantic richness at a low cost, but they lack direct depth sensing and are sensitive to environmental conditions [2,29]. Stereo systems incorporate depth information but are still vulnerable to lighting and texture challenges [16,29]. Infrared cameras are advantageous in darkness but typically offer lower resolution, while sonar sensors are reliable for close-range detection yet inadequate for detailed 3D mapping [2]. Infrared and sonar sensors are often used in Unmanned Aerial Vehicles or as complementary components rather than as primary sensors for object detection in autonomous vehicles.

For long-range and high-precision sensing, radar and LiDAR are the primary choices. Radar ensures reliable detection under adverse weather while providing velocity information via the Doppler effect [33]. LiDAR generates highly detailed 3D spatial data, but at a higher cost and with some sensitivity to atmospheric disturbances [16,29].

Although actual computational cost depends on the specific algorithms used, considering the most commonly adopted methods, sensors can be broadly classified by the computational cost they impose. Monocular and stereo cameras typically incur high computational costs due to high-resolution image processing, making real-time edge-device deployment more challenging, whereas sensors such as sonar, radar, and LiDAR often impose lower-to-medium computational overhead, better suiting resource-constrained applications.

In the context of AVs, the most common perception stack combines cameras, radar, and LiDAR. Sensor fusion is, therefore, crucial for merging semantic understanding, thereby maximizing robustness against adverse conditions and the advantages of each sensor [16]. Reliable sensor fusion demands precise calibration [29] and careful balancing between cost, complexity, and robustness. By integrating complementary strengths, fusion also introduces redundancy, essential for ensuring safe autonomous operation [33,34].

## 3. Datasets and Evaluation Metrics

### 3.1. Benchmark Datasets

Numerous datasets have been developed to support the training and evaluation of DL models in CV. These benchmarks have shaped the progression of the field by enabling more diverse training as well as standardized comparisons between methods. In what follows, the most relevant datasets for this problem are reviewed. Most of these datasets were collected in AD scenarios by equipping vehicles with multi-modal sensors and manually annotating the collected data [18].

KITTI [28] was among the first 3D OD datasets and remains widely used due to its accessibility and standardized evaluation protocols. It features real-world driving data captured in Germany using stereo cameras and a Velodyne LiDAR sensor. More recent datasets, such as nuScenes [36] and Waymo Open [37], offer greater scene diversity, richer annotations, and broader environmental conditions. nuScenes includes multi-modal data from six cameras, one LiDAR and five radars, covering 1000 driving scenes. The Waymo Open Dataset provides synchronized data from five LiDARs and five cameras, with over 230,000 annotated frames.

Although KITTI played a foundational role in the development of 3D perception methods, its limited scale and lack of environmental variety have driven a shift toward more comprehensive datasets such as nuScenes and Waymo. These newer benchmarks better support real-world deployment by exposing models to rare events, night-time conditions, and adverse weather scenarios. Due to their scale and greater diversity, nuScenes and Waymo are currently the most commonly used datasets. Nevertheless, class imbalance remains a common issue, reflecting the natural distribution of object types in driving environments [16].

Other notable datasets include ApolloScape [38], ArgoVerse [39], Lyft Level 5 [40], and H3D [41]. Table 2 summarizes the main characteristics of these datasets, based on information from [20,24,26,42].

**KITTI:** The KITTI dataset (https://www.cvlibs.net/datasets/kitti/, accessed on 25 April 2025), developed by the Karlsruhe Institute of Technology and Toyota Technological Institute at Chicago, remains one of the most widely used benchmarks for AD. It provides stereo RGB images, LiDAR PCs, and calibration files. The dataset includes 7481 training and 7518 testing frames, with over 80,000 annotated 3D bounding boxes. Objects are labelled as easy, moderate, or hard based on occlusion, truncation, and object size [29]. Data were collected using a Velodyne LiDAR, stereo cameras, and GNSS/IMU sensors across 22 scenes in urban and highway environments [32].

**nuScenes:** The nuScenes dataset (https://www.nuscenes.org/, accessed on 25 April 2025), developed by Motional (https://motional.com/), comprises 1000 20-s driving scenes recorded at 2 Hz. Each scene contains annotations for 23 categories. The sensor suite includes six cameras, a 32-beam LiDAR, and five radars. In total, the dataset features over 390,000 LiDAR sweeps and 1.4 million annotated bounding boxes [24].

**Waymo Open:** The Waymo Open Dataset (https://waymo.com/open/, accessed on 25 April 2025) includes approximately 230,000 annotated frames and over 12 million 3D bounding boxes. It provides synchronized data from five LiDAR sensors and five cameras, spanning 798 training, 202 validation, and 150 test segments. Annotated classes cover vehicles, pedestrians, cyclists, and traffic signs [24].

### 3.2. Evaluation Metrics

#### 3.2.1. General Metrics

To quantitatively evaluate 3D OD methods, a variety of evaluation metrics have been proposed. These metrics extend foundational concepts from 2D OD by incorporating depth, orientation, and volumetric estimation. While the core ideas, such as precision, recall, and average precision (AP), remain central, they are adapted to operate on 3D bounding boxes.

The major difference between 2D and 3D detection metrics lies in the matching criteria between ground truths and predictions when calculating precision and recall. The quality of a detection is commonly measured using the Intersection over Union (IoU), which computes the ratio of the overlapping volume between a predicted and ground-truth 3D bounding box. A prediction is typically considered a true positive (TP) if the IoU exceeds a predefined threshold (usually 0.5); otherwise, it is classified as a false positive (FP). Missed detections are treated as false negatives (FNs), and correctly ignored negatives are true negatives (TNs). These categories form the basis of the confusion matrix:

- **True Positives** (TP): Correctly predicted positives.
- **False Positives** (FP): Incorrectly predicted positives.
- **False Negatives** (FN): Missed predictions.
- **True Negatives** (TN): Correctly predicted negatives.

Based on these, two key performance metrics are defined:

(3) $$\mathrm{Precision}=\frac{TP}{TP+FP},$$

(4) $$\mathrm{Recall}=\frac{TP}{TP+FN}.$$

Precision quantifies the correctness of predicted positives, while recall captures the detector’s ability to find all relevant objects. These metrics are often visualized through precision–recall (P-R) curves, from which the average precision (AP) is derived. In theory, both precision and recall should be as close to 1 (one) as possible. However, in practice, they often conflict; improving one may degrade the other.

The AP for a given class is computed as the area under the P-R curve:

(5) $$AP=\int_{0}^{1}\max\left\{p\left(r\right)|r\geq r^{\prime }\right\}\;dr,$$

where p(r) is the precision at recall *r*.

Once the AP is computed for each object class, the mean average precision (mAP) is obtained by averaging across all classes. The setting of the IoU threshold plays a critical role here, as it affects the strictness and comprehensiveness of the evaluation. For instance, the COCO benchmark computes mAP over multiple IoU thresholds, ranging from 0.5 to 0.95 in steps of 0.05, to provide a robust evaluation of localization and classification performance.

IoU is computed using the volumes of the bounding boxes:

(6) $$\mathrm{IoU}=\frac{\left|A\cap B\right|}{\left|A\cup B\right|},$$

where *A* and *B* represent the predicted and ground-truth 3D bounding boxes, respectively. This volumetric IoU is more sensitive to spatial misalignment than its 2D counterpart, making evaluation more stringent.

While AP and mAP remain the principal metrics for assessing detection quality, additional metrics are crucial in real-time or resource-constrained scenarios. These include the number of model parameters and floating-point operations per second (FLOPs), which reflect computational complexity, as well as latency and frames per second (FPS), which assess runtime efficiency. Together, these metrics offer a holistic view of a model’s accuracy, efficiency, and deployability.

#### 3.2.2. Dataset-Specific Metrics

**KITTI:** The KITTI benchmark [28] remains one of the most commonly used datasets for 3D OD. It evaluates model performance using three primary metrics [16]:

- **AP_2D_**: Average precision computed by projecting the predicted 3D bounding boxes into the 2D image plane and calculating 2D IoU.
- **AP_3D_**: Average precision computed using the full 3D bounding box IoU.
- **AP_BEV_**: Average precision computed from a bird’s-eye view (BEV) projection of the 3D bounding box.

In addition to these metrics, KITTI also reports the Average Orientation Similarity (AOS), which assesses the alignment between predicted and ground-truth object orientations. Different object classes have distinct IoU thresholds for evaluation: for instance, 0.7 for cars and 0.5 for pedestrians and cyclists. This accounts for the relative difficulty of localizing small or occluded objects.

**nuScenes:** The nuScenes benchmark [36] proposes a more comprehensive evaluation scheme that moves beyond traditional IoU-based matching. The authors argue that using IoU alone does not capture all relevant aspects of detection quality in complex urban environments. Instead, nuScenes introduces centre-based matching, where predicted objects are associated with the ground truth based on their 2D centre distance on the ground plane. The newly introduced scores quantify how closely the predicted objects align with the ground truth not just in terms of location, but also shape, pose, and dynamic behaviour. The final nuScenes Detection Score (NDS) aggregates the mean average precision (mAP) and the mean TP metrics (mTP) into a single holistic score:

(7) $$NDS=\frac{1}{10}\left(5\cdot mAP+\sum_{i=1}^{5}\left(1-\min\left(1,mTP_{i}\right)\right)\right)$$

This score captures both detection accuracy and multi-attribute consistency, making it a more holistic evaluation metric.

**Waymo Open Dataset**: The Waymo benchmark [37] evaluates detection at two levels:

- **Level 1 (L1)**: Objects with at least five LiDAR points inside the bounding box.
- **Level 2 (L2)**: All annotated objects, including sparse detections.

To capture different aspects of model performance, the dataset defines multiple variants of average precision. The standard AP measures detection accuracy using IoU-based matching, while APH extends this by incorporating heading angle accuracy, evaluating how well the model predicts object orientation. Additionally, Waymo introduces a variant based on the Hungarian algorithm, which performs optimal one-to-one assignment between predictions and ground truth, particularly beneficial when multiple nearby detections could otherwise result in ambiguity.

## 4. Taxonomy and Review

Artificial Intelligence (AI), and in particular neural networks (NNs), have become foundational to many modern technological applications. DL, a subfield of ML and AI, enables models to learn hierarchical and abstract representations from large volumes of data, significantly improving prediction accuracy across various domains. Its flexibility and high capacity for pattern recognition have made DL especially valuable in AVs, where many SoA systems rely heavily on deep CNNs.

CNNs have demonstrated exceptional performance in 2D image-based tasks, including classification and object detection. However, their extension to 3D perception, especially OD in PCs, introduces specific challenges. Unlike structured image grids, PCs are inherently unordered, irregular, and sparse, making standard convolution operations unsuitable without significant modification. Specialized architectures and preprocessing techniques are thus required to adapt CNN-based methods to 3D inputs.

The widespread adoption of DL has been driven by two key enablers: the availability of large-scale annotated datasets and advances in Graphics Processing Unit (GPU) hardware, which support high-throughput parallel computation [31]. DL models are composed of multiple layers of artificial neurons that transform input data using learnable weights and biases. These weights are optimized during training by minimizing a loss function that quantifies the error between predictions and ground truth [27]. The methods reviewed in this work are primarily based on supervised learning, where models are trained using labelled datasets to learn relevant spatial and semantic patterns.

This section presents an overview of the main families of 3D OD approaches, organized according to the type of sensor input and processing strategy. For each modality or hybrid configuration, we highlight the key trends and most influential methods that have shaped the field.

### 4.1. Taxonomy of 3D Object Detection

Compared to 2D OD, 3D OD introduces distinct challenges, particularly in terms of depth perception and spatial representation. When working with image data alone, the primary limitation is the lack of explicit depth information. Two common strategies are used to address this: (i) decoupling the task into 2D OD followed by depth estimation, often using geometric constraints or priors to reconstruct 3D positions; and (ii) employing DL models that directly infer 3D object properties from image inputs. In this work, we focus on the latter approach, where monocular, stereo, and multi-view camera configurations are used to extract depth-aware features through learned representations.

In contrast, when only PC data are available, typically acquired using LiDAR, the main challenge becomes learning from data that are sparse, unordered, and non-uniform. Various strategies have emerged to process such data effectively, and these are commonly grouped into four categories: point-based methods, which operate directly on raw PC coordinates; voxel-based methods, which discretize the space into regular 3D grids; point–voxel hybrid approaches, which combine the advantages of both representations; and projection-based methods, which transform 3D data into 2D representations to leverage mature 2D CNN architectures.

Radar-based 3D detection methods are also gaining attention due to radar’s robustness in adverse weather and lighting conditions. However, the relatively low spatial resolution and noisy measurements of radar data pose unique challenges. Most radar-based approaches either project radar points into bird’s-eye view representations or fuse them with other modalities to compensate for their limitations.

Multi-modal fusion methods attempt to harness the complementary strengths of different sensor modalities, most commonly combining camera and LiDAR data. These methods are generally categorized based on the stage at which the fusion occurs within the perception pipeline: early fusion combines raw sensor data before feature extraction; mid-level fusion integrates intermediate features from each modality; and late fusion merges the outputs of independent modality-specific detectors.

**Figure 4:**
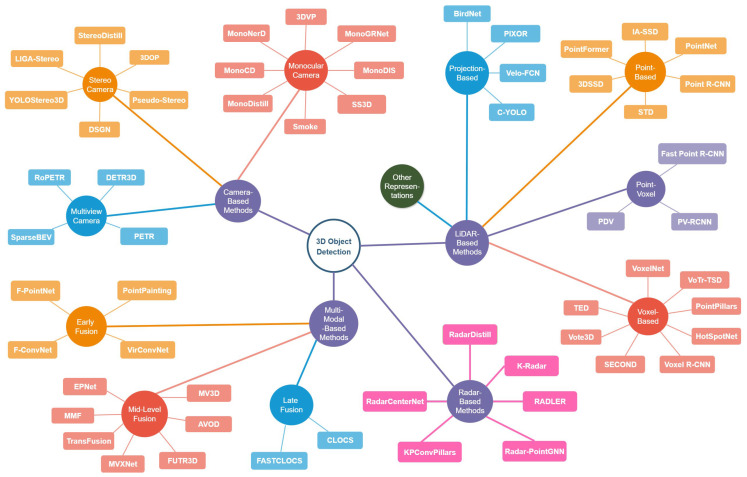
Taxonomy of 3D object detection methods categorized by input modality.

Although there is some agreement in the literature regarding the categorization of 3D OD methods, variations in terminology and classification criteria remain. To address this, a high-level taxonomy was developed to capture the dominant and most widely adopted strategies. Figure 4 illustrates the proposed taxonomy and maps out the representative methods covered in this review.

### 4.2. Camera-Based Methods

Three-dimensional OD methods often build upon principles inherited from 2D detectors. However, the transition from 2D to 3D introduces increased sensitivity to localization errors. While a few pixels of deviation in 2D detection may still result in acceptable IoU scores, small spatial misalignments in 3D, even at the decimetre scale, can significantly degrade detection accuracy. This section presents the most prominent camera-based strategies for 3D OD, including monocular, stereo, and multi-view or multi-camera approaches.

#### 4.2.1. Monocular-Based Methods

Monocular 3D OD aims to predict object depth, orientation, and size using only a single RGB image. Its minimal hardware requirements make it attractive for large-scale deployment, particularly in cost-sensitive or lightweight platforms [29].

One of the earliest works, 3DVP [43], introduced a viewpoint-aware classification model that leveraged sub-category structures. Mono3D [44] advanced the field by generating 3D proposals on a learned ground plane, providing a robust geometry-aware baseline. Deep3DBox [45] streamlined the pipeline with a single network predicting object orientation and dimensions end-to-end.

Subsequent efforts focused on improving both accuracy and efficiency. M3D-RPN [46] was the first to introduce a two-stage Region Proposal Network tailored for monocular 3D detection. MonoDIS [47] disentangled depth, shape, and orientation learning into separate branches, improving training stability. Lightweight single-shot architectures like SS3D [48] and SMOKE [49] removed the need for dense proposals, achieving real-time inference.

Recent developments leverage auxiliary knowledge sources. MonoDistill [50] and MonoSKD [51] showed how student models can benefit from distilled supervision by larger teacher networks. MonoNeRD [52] incorporated neural radiance field priors to model fine-grained object appearance and geometry. MonoCD [53] achieved SoA results without relying on LiDAR or dense proposal mechanisms.

#### 4.2.2. Stereo-Based Methods

Stereo-based 3D OD estimates depth using the disparity between stereo image pairs. The resulting depth maps are fused with 2D detection outputs to localize objects in 3D. Compared to monocular approaches, stereo methods benefit from geometric priors and disparity cues that enable more accurate depth perception [19,24].

Early approaches like 3DOP relied on hand-crafted features and sliding-window proposals. Stereo R-CNN [54] marked a shift toward DL-based stereo detection by integrating a disparity-aware ROI head into a Faster R-CNN backbone. RT3D-Stereo further improved efficiency by introducing a lightweight cost volume and a 3D-aware regression module.

DSGN [55] constructed a scene-level representation over stereo cost volumes and refined object predictions using message passing. YOLOStereo3D [56] adopted a single-shot architecture to streamline the detection process. LIGA-Stereo [57] combined sparse LiDAR signals with stereo features to guide attention. More recently, StereoDistill [58] demonstrated that compact student networks can replicate the performance of larger stereo teacher models through structured distillation.

#### 4.2.3. Multi-View/Multi-Camera-Based Methods

Multi-camera systems are frequently used in AVs to provide 360° perception of the environment. These methods aggregate multiple views to construct a unified 3D scene representation, often by projecting image features into a bird’s-eye view (BEV) space [19]. However, this projection introduces ambiguity due to the absence of explicit depth information, which complicates accurate feature alignment.

To mitigate this, recent approaches have adopted transformer-based architectures that enhance spatial reasoning and cross-view feature fusion. DETR3D [59] introduced 3D object queries that attend to relevant regions across all views. PETR [60] enriched positional encoding to improve object localization. BEVFormer [61] leveraged temporal consistency across video frames to enhance BEV features. SparseBEV [62] and RoPETR [63] proposed attention-efficient alternatives to reduce computational cost while maintaining high spatial fidelity.

#### 4.2.4. Discussion

Camera-based methods are attractive due to their affordability and ability to capture rich semantic features such as colour, texture, and appearance. Many 3D detectors for camera inputs borrow architectural principles from well-established 2D detection models [32]. However, monocular methods continue to face performance limitations. Small depth estimation errors can lead to significant inaccuracies in 3D localization, which is particularly problematic in safety-critical scenarios like AVs.

To address these limitations, pseudo-LiDAR methods have been proposed, which convert estimated depth into synthetic point clouds. While this approach brings monocular setups closer to LiDAR-like reasoning, its performance remains constrained by depth prediction quality and calibration accuracy. Stereo methods offer improved depth estimation through geometric constraints but require high computational resources for disparity matching and cost volume construction.

Multi-view approaches enable comprehensive scene understanding by leveraging multiple overlapping views. However, without depth supervision, aligning features across views remains a key challenge. Transformer-based methods have made significant progress in this area, particularly in benchmarks like nuScenes that provide complete camera coverage. These models continue to evolve toward more efficient cross-view fusion and real-time inference.

In terms of evaluation, traditional 2D metrics such as AP_*2D*_ or orientation similarity scores are insufficient to fully assess 3D detection performance. The most relevant metric for these models is AP_*3D*_, often accompanied by AP_*BEV*_ to capture localization accuracy in the ground plane. To support fair comparison, Table 3 summarizes the performance of key camera-based 3D detectors, reporting AP_*2D*_, AP_*3D*_, and AP_*BEV*_ on KITTI (for easy (E), moderate (M), and hard (H) difficulty levels), as well as mAP and NDS on nuScenes, and L1/L2 mAP on Waymo. Inference times and hardware details are also included.

### 4.3. LiDAR-Based Methods

Unlike images, where pixels are regularly distributed and can be processed using conventional CNNs, PCs are sparse, unordered, and irregular 3D representations. LiDAR sensors generate such PCs by emitting laser pulses and recording their return time, capturing the high-resolution geometric structure of the environment with strong spatial accuracy [64]. However, this structure introduces unique challenges for DL models, which are typically designed for dense and grid-like data.

Traditional LiDAR processing relied on geometric and topological cues to extract object-level information, for example by detecting abrupt changes in surface orientation or reflectivity to infer object boundaries [65]. While computationally efficient and interpretable, such classical methods often struggle in complex scenes with occlusions, clutter, or sparse measurements.

Modern DL approaches, by contrast, have been built around more structured or learned representations of the PC. To make LiDAR data compatible with NN architectures, the raw points are typically transformed into one of several possible intermediate formats. Most works in the literature categorize these into four main classes: point-based, voxel-based, hybrid point–voxel, and projection-based representations. Other alternatives, such as range-view, graph-based, and transformer-based models, have also emerged. Each representation encodes spatial information differently, leading to trade-offs in runtime, memory consumption, and geometric fidelity.

#### 4.3.1. Projection-Based Methods

Projection-based methods convert 3D point clouds into 2D representations to leverage efficient and mature 2D CNNs. Common projection types include front view, spherical view, and most prominently, BEV, which projects points onto the xy-plane. BEV is especially popular in AD due to the constrained ground motion of vehicles.

BEV reduces occlusions, enables consistent metric scaling, and avoids overlapping object bounding boxes. The process usually involves discretizing *x* and *y* positions into pixel grids, while collapsing the height dimension [66]. The resulting pseudo-image encodes statistics such as point density, intensity, and height per cell.

Early works like PIXOR [67], Complex-YOLO [68], and BirdNet [69] adopted standard 2D CNNs to process BEV inputs, showing the feasibility of this approach. Complex-YOLO refined anchor definitions and introduced orientation modelling in polar coordinates. Despite their speed and simplicity, projection-based methods discard vertical detail and are sensitive to sparsity and resolution settings. Nonetheless, they offer an attractive balance between speed and accuracy in real-time scenarios.

#### 4.3.2. Point-Based Methods

Point-based methods operate directly on raw 3D coordinates without intermediate discretization. These approaches preserve fine-grained geometry and avoid quantization artefacts, but must account for the unordered nature of point sets.

PointNet [70] was a foundational work in this space, introducing permutation-invariant architectures via shared MLPs and symmetric aggregation functions. However, it mainly captured global context, limiting its performance on local geometric structures. PointNet++ [71] addressed this limitation through hierarchical feature extraction on local neighbourhoods.

Subsequent methods such as PointRCNN [72], 3DSSD, STD, IA-SSD [73], and PointFormer [74] improved accuracy by incorporating attention mechanisms, local spatial encoding, and direct 3D box regression. These models maintain high geometric fidelity and excel in preserving object boundaries. However, they often involve expensive neighbourhood search operations and are computationally demanding, making real-time deployment more difficult.

#### 4.3.3. Voxel-Based Methods

Voxel-based methods discretize the 3D space into a structured grid of equally sized volumetric elements (voxels), allowing the application of 3D convolutions. Each voxel can encode occupancy, intensity, and local statistics. While this enables efficient parallel processing, voxelization introduces resolution–memory trade-offs and can result in the loss of fine geometric detail.

VoxelNet [75] was the first end-to-end model to apply voxel-based feature learning. SECOND [76] introduced sparse 3D convolutions to reduce computational cost, while PointPillars [77] collapsed the vertical dimension, forming pseudo-pillars to enable fast 2D convolution on BEV-like features.

More recent voxel-based models have incorporated transformer architectures and attention mechanisms to improve performance. HotSpotNet [78], Voxel R-CNN [79], VoTr-TSD [80], and TED [81] represent this line of progress. TED currently holds state-of-the-art performance among LiDAR-only methods on the KITTI benchmark. These methods strike a balance between regular structure and accuracy but remain constrained by cubic memory growth with increasing resolution. Notably, only around 1% of voxels are occupied in KITTI and 3% in Waymo [16], highlighting inefficiencies in voxel-based representations.

#### 4.3.4. Point–Voxel Hybrid Methods

Hybrid methods aim to combine the geometric richness of point-based models with the efficiency of voxel-based networks. Typically, voxelized backbones are used for coarse region proposals, which are then refined using point-level features for more precise bounding box prediction.

Fast Point R-CNN [82], PV-RCNN [83], and PDV [84] exemplify this family of detectors. These models perform repeated point-to-voxel and voxel-to-point transformations, enabling fine-grained multi-scale feature fusion. While they achieve high accuracy and robust object boundary modelling, their bidirectional feature interaction comes at the cost of additional memory usage and runtime complexity.

#### 4.3.5. Other Representations

Beyond the dominant four categories, other representations have emerged. Range-based methods treat LiDAR scans as 2D range images, enabling the use of efficient 2D convolutions [70]. Graph-based methods model PCs as node–edge structures to capture spatial topology. More recently, transformer-based architectures have gained traction for their ability to model long-range dependencies and support parallel computation, making them increasingly popular in AD perception pipelines [85].

#### 4.3.6. Discussion

LiDAR-based 3D OD has matured significantly, supported by diverse representations and architectural innovations. Each strategy introduces specific advantages and trade-offs.

Point-based models preserve raw geometry and deliver precise localization but remain computationally demanding and hard to scale to large scenes. Voxel-based models offer regular structure and efficient parallelization, but their memory usage scales cubically with resolution and suffers from data sparsity. Projection-based approaches enable real-time processing via 2D CNNs and are dominant in deployment settings, though their performance is bounded by the quality of projection and resolution settings. Hybrid methods strike a strong accuracy–runtime balance, but their complexity and synchronization cost limit scalability.

Ultimately, the choice of representation depends on the application: point-based for highest fidelity, voxel- or projection-based for efficient real-time inference, and hybrid models for balanced performance. A summary of the most representative LiDAR-based detection models, categorized by representation and benchmark performance, is provided in Table 4. This enables direct comparison with camera-based approaches in Table 3, and illustrates how architectural choices impact detection performance across the chosen datasets.

### 4.4. Radar-Based Methods

Unlike vision-based systems, radar offers consistent performance in challenging environmental conditions, making it well-suited for 3D object detection. The first DL approach, Radar-PointGNN (2021) [86], modelled the sparse range–Doppler point cloud as a graph and applied graph neural networks to capture spatial relationships. In 2022, K-Radar [87] introduced a grid-based fusion of multi-view radar snippets, enabling dense feature extraction across time, and KPConvPillars [88] adapted the KPConv convolution to radar pillars, significantly improving detection resolution and angular precision.

More recent methods leverage teacher–student distillation and large-scale pretraining to overcome data scarcity. RadarDistill (2024) [89] distilled LiDAR-based feature representations into a radar-only student network, achieving substantial gains in both range and velocity estimation. In 2025, RADLER [90] presented a multimodal foundation model that pretrains on synchronized radar–camera–LiDAR streams, demonstrating that rich cross-modal pretraining can elevate pure radar detection performance to higher levels of performance. Table 5 aggregates some of the most important radar-based methods compared under the few values reported in benchmarked datasets.

### 4.5. Multi-Modal-Based Methods

Multi-modal methods integrate multiple sensor modalities to exploit their complementary characteristics in 3D OD. The most prevalent fusion strategy combines LiDAR point clouds and RGB images, as these are the primary sensors in autonomous AD platforms. While LiDAR provides accurate geometric structure in 3D, images offer dense semantic and texture rich cues. When fused effectively, these modalities compensate for each other’s limitations, such as occlusions, sparse depth, or poor lighting, and improve detection robustness [24,32].

Single-modality systems are often insufficient for real-world deployment. Cameras, while rich in appearance information, lack direct depth perception. Conversely, LiDAR sensors offer spatial accuracy but no visual appearance or colour. Fusion thus enhances the overall reliability and accuracy of perception systems, particularly in adverse conditions or sensor failures. Most autonomous vehicles are equipped with both cameras and LiDAR, making sensor fusion a practical and effective design choice.

Fusion can be performed at different stages of the detection pipeline, broadly categorized into three levels [29,32]: *early fusion*, where raw or low-level features are combined; *mid-level fusion*, where intermediate features are merged after being learned independently; and *late fusion*, where modality-specific decisions are integrated at the final stage.

#### 4.5.1. Early Fusion Methods

Early fusion strategies integrate raw sensor inputs or low-level features before any deep processing. A common approach is to project LiDAR points onto the image plane and enhance them with pixel-level semantic information. For instance, PointPainting [91] enriches LiDAR points with semantic segmentation scores from an image-based network, effectively augmenting 3D input with 2D semantic priors. Similarly, Frustum PointNet [92] first uses 2D detectors to define frustums in 3D space and then applies a point-based network to the subset of points within each frustum. This pipeline was further extended by F-ConvNet [93] and Frustum PointPillars [94], which replace the 3D backbone to improve detection. More recently, VirConvNet [95] has achieved SoA performance on KITTI by employing virtual convolutions across fused representations.

While early fusion offers tight cross-modal interaction and benefits from direct semantic guidance, it is highly sensitive to calibration errors and temporal misalignment. The requirement for accurate extrinsic and intrinsic calibration means any deviation can degrade performance. Additionally, the preprocessing stages, including 2D segmentation or detection, add computational overhead and increase latency during both training and inference.

#### 4.5.2. Mid-Level Fusion Methods

Mid-level, or deep fusion, combines intermediate features extracted independently from each sensor modality. These features are often spatially aligned in a common space, typically BEV, frustum, or image views, before fusion. This allows the network to learn cross-modal interactions while retaining modality-specific inductive biases.

MV3D [96] was one of the earliest examples, combining features from LiDAR BEV, LiDAR front view, and image view during the proposal refinement stage. AVOD [97] extended this by integrating LiDAR and image features for both proposal generation and refinement. ContFuse [98] introduced continuous convolutions to merge multi-scale features in a pixel-wise manner, although its performance can degrade with sparse or noisy inputs. MMF [99] incorporated auxiliary tasks such as ground plane estimation and depth completion to improve feature fusion robustness.

Later models introduced more advanced fusion mechanisms. EPNet [100] constructs image-aware proposal features by conditioning LiDAR features on image context. Transformer-based methods such as TransFusion [101] and FUTR3D [102] introduced cross-modal attention modules, improving feature alignment and information flow across views and modalities.

Mid-level fusion offers a compelling balance between semantic richness and spatial precision. Unlike early fusion, it avoids the need for raw data alignment, and unlike late fusion, it allows rich interaction between feature spaces. However, aligning intermediate features remains challenging due to differing receptive fields, sampling rates, and spatial resolutions across modalities.

#### 4.5.3. Late Fusion Methods

Late fusion approaches process each modality independently through separate detection branches and merge their outputs during post-processing. This may involve strategies such as confidence-weighted fusion, geometric consistency checks, or non-maximum suppression (NMS) across modalities.

CLOCs [103] represents a modular late-fusion system that refines and merges 2D and 3D detections based on semantic and geometric constraints. Fast-CLOCs [104] optimized this pipeline for real-time deployment. While late fusion is computationally efficient and allows for reuse of pretrained single-modality detectors, it lacks the fine-grained interaction and synergy of feature-level fusion. As a result, its performance tends to lag behind early and mid-level methods.

#### 4.5.4. Discussion

Multi-modal fusion consistently achieves SoA performance by exploiting the strengths of each sensor: LiDAR ensures precise spatial localization, while images enrich detections with contextual and semantic information. Fusion pipelines are often LiDAR-centric, using LiDAR data to generate proposals and images to refine object categories or adjust box parameters. This configuration has become the dominant architecture in modern systems.

However, fusion introduces its own set of challenges. Misalignment due to unsynchronized timestamps, calibration drift, and different acquisition frequencies can lead to incorrect correspondences between modalities. Resolution mismatches are also a concern, as many LiDAR points may fall into a single pixel, and vice versa. Additionally, some objects may be visible in only one modality, creating further ambiguity during training and inference. Effective data augmentation must be jointly applied across sensors to avoid inconsistencies [24,29].

While camera–LiDAR fusion dominates the literature, radar has gained attention as a complementary sensor. Radar systems are robust under adverse weather, offer long-range detection, and can directly measure velocity. However, radar data are typically sparse and noisy with poor spatial resolution, making stand-alone 3D detection difficult. Moreover, the lack of large-scale radar datasets has limited its development. Instead, radar is usually fused with LiDAR or image data.

Fusion methods like RadarNet [105] and RICCARDO [106] incorporate radar and camera data, while frameworks such as CenterFusion [107] explore the fusion of LiDAR and radar data. Surveys like [108] for camera–radar fusion and [109] for LiDAR–radar fusion provide greater detail in these types of methods. Despite its potential, radar fusion remains underexplored compared to camera–LiDAR fusion and is often used as a supplementary modality.

Table 6 summarizes the most representative fusion-based 3D OD models, categorized by fusion strategy and sensor combination. It includes detection performance across benchmarks such as KITTI and nuScenes, as well as inference runtimes and year of publication. This taxonomy complements the earlier tables, allowing a systematic comparison of fusion approaches against LiDAR-only and camera-based detectors.

**Table 3:** Camera-based 3D object detection results on KITTI car test set, nuScenes test set, and Waymo validation set (for easy (E), moderate (M) and hard (H) difficulty levels).

Method	Year	AP_2D_			AP_3D_			AP_BEV_			nuScenes		Waymo		Time (s)	Hardware	Code Available
		E	M	H	E	M	H	E	M	H	mAP	NDS	L1 mAP	L2 mAP			
***Monocular Camera:***																	
3DVP [43]	2015	84.95	76.98	65.78	–	–	–	–	–	–	–	–	–	–	40	8 cores @ 3.5 GHz (Matlab + C/C++)	✓
Mono3D [44]	2016	80.30	67.29	62.23	–	–	–	–	–	–	–	–	–	–	4.2	GPU @ 2.5 GHz (Matlab + C/C++)	✓
SubCNN [110]	2016	94.26	89.98	79.78	–	–	–	–	–	–	–	–	–	–	2	GPU @ 3.5 GHz (Python + C/C++)	✓
Deep3DBox [45]	2016	94.71	90.19	76.82	–	–	–	–	–	–	–	–	–	–	1.5	GPU @ 2.5 GHz (C/C++)	✓
Deep MANTA [111]	2017	98.89	93.50	83.21	–	–	–	–	–	–	–	–	–	–	0.7	GPU @ 2.5 GHz (Python + C/C++)	×
3D-RCNN [112]	2018	90.02	89.39	80.29	–	–	–	–	–	–	–	–	–	–	–	–	✓
ROI-10D [113]	2018	76.56	70.16	61.15	4.32	2.02	1.46	9.78	4.91	3.74	–	–	–	–	0.20	GPU @ 3.5 GHz (Python)	×
MF3D [114]	2018	90.43	87.33	76.78	7.08	5.18	4.68	13.73	9.62	8.22	–	–	–	–	–	–	✓
MonoGRNet [115]	2018	88.65	77.94	63.31	9.61	5.74	4.25	18.19	11.17	8.73	–	–	–	–	0.04	NVIDIA P40	✓
GS3D [116]	2019	86.23	76.35	62.67	4.47	2.90	2.47	8.41	6.08	4.94	–	–	–	–	2	1 core @ 2.5 GHz (C/C++)	×
Mono3D-PLiDAR [117]	2019	80.85	53.36	44.80	10.76	7.50	6.10	21.27	13.92	11.25	–	–	–	–	0.10	NVIDIA GeForce 1080 (pytorch)	×
AM3D [118]	2019	92.55	88.71	77.88	16.50	10.74	9.52	27.91	22.24	18.62	–	–	–	–	0.40	GPU @ 2.5 GHz (Python + C/C++)	×
Deep Optics [119]	2019	–	–	–	16.86	13.82	13.26	26.71	19.87	19.11	–	–	–	–	–	–	×
CenterNet [120]	2019	–	–	–	–	–	–	–	–	–	33.80	40.00	–	–	–	–	✓
FQNet [121]	2019	94.72	90.17	76.78	2.77	1.51	1.01	5.40	3.23	2.46	–	–	–	–	0.50	1 core @ 2.5 GHz (Python)	×
Shift R-CNN [122]	2019	94.07	88.48	78.34	6.88	3.87	2.83	11.84	6.82	5.27	–	–	–	–	0.25	GPU @ 1.5 GHz (Python)	×
MonoFENet [123]	2019	91.68	86.63	76.71	8.35	5.14	4.10	17.03	11.03	9.05	–	–	–	–	0.15	1 core @ 3.5 GHz (Python)	×
MonoDIS [47]	2019	90.31	87.58	76.85	10.37	7.94	6.40	18.80	19.08	17.41	30.40	38.40	–	–	–	–	✓
MonoPSR [124]	2019	93.63	88.50	73.36	10.76	7.25	5.85	18.33	12.58	9.91	–	–	–	–	0.20	GPU @ 3.5 GHz (Python)	✓
MoVi-3D [125]	2019	–	–	–	15.19	10.90	9.26	22.76	17.03	14.85	–	–	–	–	–	–	×
RefinedMPL [126]	2019	88.29	65.24	53.20	18.09	11.14	8.94	28.08	17.60	13.95	–	–	–	–	0.15	GPU @ 2.5 GHz (Python + C/C++)	×
M3D-RPN [46]	2019	89.04	85.08	69.26	14.76	9.71	7.42	21.02	13.67	10.23	–	–	–	–	0.16	GPU @ 1.5 GHz (Python)	✓
SS3D [48]	2020	92.72	84.92	70.35	10.78	7.68	6.51	16.33	11.52	9.93	–	–	–	–	0.048	Tesla V100	✓
MonoPair [127]	2020	96.61	93.55	83.55	13.04	9.99	8.65	19.28	14.83	12.89	–	–	–	–	0.06	GPU @ 2.5 GHz (Python + C/C++)	×
RTM3D [128]	2020	91.82	86.93	77.41	14.41	10.34	8.77	19.17	14.20	11.99	–	–	–	–	0.05	GPU @ 1.0 GHz (Python)	✓
SMOKE [49]	2020	93.21	87.51	77.66	14.03	9.76	7.84	20.83	14.49	12.75	–	–	–	–	0.03	GPU @ 2.5 GHz (Python)	✓
PatchNet [129]	2020	–	–	–	15.68	11.12	10.17	22.97	16.86	14.97	–	–	0.39	0.38	0.4	1 core @ 2.5 GHz (C/C++)	×
IAFA [130]	2020	93.08	89.46	79.83	17.81	12.01	10.61	25.88	17.88	15.35	–	–	–	–	0.04	1 core @ 2.5 GHz (C/C++)	×
Kinematic3D [131]	2020	89.67	71.73	54.97	19.07	12.72	9.17	26.69	17.52	13.10	–	–	–	–	0.12	1 core @ 1.5 GHz (C/C++)	✓
KM3D [132]	2020	96.44	91.07	81.19	16.73	11.45	9.92	23.44	16.20	14.47	–	–	–	–	0.03	1 core @ 2.5 GHz (Python)	✓
DDMP-3D [133]	2021	91.15	81.70	63.12	19.71	12.78	9.80	28.08	17.89	13.44	–	–	–	–	0.18	1 core @ 2.5 GHz (Python)	✓
MonoRUn [134]	2021	95.48	87.91	78.10	19.65	12.30	10.58	27.94	17.34	15.24	–	–	–	–	0.07	GPU @ 2.5 GHz (Python + C/C++)	✓
GrooMeD-NMS [135]	2021	90.14	80.28	63.78	18.10	12.32	9.65	26.19	18.27	14.05	–	–	–	–	0.12	1 core @ 2.5 GHz (Python)	✓
MonoDLE [136]	2021	93.83	90.81	80.93	17.23	12.26	10.29	24.79	18.89	16.00	–	–	–	–	0.04	GPU @ 2.5 GHz (Python)	✓
CaDDN [137]	2021	93.61	80.73	71.09	19.17	13.41	11.46	27.94	18.91	17.19	–	–	–	–	0.63	GPU @ 2.5 GHz (Python)	✓
MonoFlex [138]	2021	96.01	91.02	83.38	19.94	13.89	12.07	28.23	19.75	16.89	–	–	–	–	0.03	GPU @ 2.5 GHz (Python)	✓
MonoRCNN [139]	2021	91.90	86.48	66.71	18.36	12.65	10.03	25.48	18.11	14.10	–	–	–	–	0.07	GPU @ 2.5 GHz (Python)	✓
FCOS3D [140]	2021	–	–	–	–	–	–	–	–	–	35.80	42.80	–	–	–	–	✓
MonoEF [141]	2021	96.32	90.88	83.27	21.29	13.87	11.71	29.03	19.70	17.26	–	–	–	–	0.03	1 core @ 2.5 GHz (Python)	✓
GUPNet [142]	2021	94.15	86.45	74.18	22.26	15.02	13.12	30.29	21.19	18.20	–	–	–	–	–	1 core @ 2.5 GHz (Python + C/C++)	✓
PGD [143]	2021	92.04	80.58	69.67	19.05	11.76	9.39	26.89	16.51	13.49	38.60	44.80	–	–	0.03	1 core @ 2.5 GHz (C/C++)	✓
Aug3D-RPN [144]	2021	85.57	77.88	61.16	17.82	12.99	9.78	26.00	17.89	14.18	–	–	–	–	0.08	1 core @ 2.5 GHz (C/C++)	×
DD3D [145]	2021	94.69	93.99	89.37	23.19	16.87	14.36	32.35	23.41	20.42	41.80	47.70	–	–	–	1 core @ 2.5 GHz (C/C++)	✓
PCT [146]	2021	96.45	88.78	78.85	21.00	13.37	11.31	29.65	19.03	15.92	–	–	0.89	0.66	0.045	1 core @ 2.5 GHz (Python)	✓
Autoshape [147]	2021	86.51	77.60	64.40	22.47	14.17	11.36	30.66	20.08	15.95	–	–	–	–	0.04	1 core @ 2.5 GHz (C/C++)	✓
DLE [148]	2021	94.66	84.45	62.10	24.23	14.33	10.30	31.09	19.05	14.13	–	–	–	–	0.06	NVIDIA Tesla V100	×
MonoCon [149]	2021	–	–	–	22.50	16.46	13.95	31.12	22.10	19.00	–	–	–	–	–	–	✓
MonoDistill [50]	2022	–	–	–	22.97	16.03	13.60	31.87	22.59	19.72	–	–	–	–	–	–	✓
MonoDTR [150]	2022	93.90	88.41	76.20	21.99	15.39	12.73	28.59	20.38	17.14	–	–	–	–	0.04	1 core @ 2.5 GHz (C/C++)	✓
MonoDETR [151]	2022	93.99	86.17	76.19	24.52	16.26	13.93	32.20	21.45	18.68	–	–	–	–	0.04	1 core @ 2.5 GHz (Python)	✓
MonoJSG [152]	2022	–	–	–	24.69	16.14	13.64	32.59	21.26	18.18	–	–	–	–	–	–	✓
HomoLoss [153]	2022	95.92	90.69	80.91	21.75	14.94	13.07	29.60	20.68	17.81	–	–	–	–	0.04	1 core @ 2.5 GHz (Python)	×
MonoDDE [154]	2022	96.76	89.19	81.60	24.93	17.14	15.10	33.58	23.46	20.37	–	–	–	–	0.04	1 core @ 2.5 GHz (Python)	×
Mix-Teaching [155]	2022	96.35	91.02	83.41	26.89	18.54	15.79	35.74	24.23	20.80	–	–	–	–	30	1 core @ 2.5 GHz (C/C++)	✓
DCD [156]	2022	96.44	90.93	83.36	23.81	15.90	13.21	32.55	21.50	18.25	–	–	–	–	0.03	1 core @ 2.5 GHz (C/C++)	✓
DEVIANT [157]	2022	94.42	86.64	76.69	21.88	14.46	11.89	29.65	20.44	17.43	–	–	–	–	0.04	1 GPU (Python)	✓
Cube R-CNN [158]	2022	95.78	92.72	84.81	23.59	15.01	12.06	31.70	21.20	18.43	–	–	–	–	0.05	GPU @ 2.5 GHz (Python)	✓
MoGDE [159]	2022	–	–	–	27.07	17.88	15.66	38.38	25.60	22.91	–	–	–	–	–	–	×
ADD [160]	2022	94.82	89.53	81.60	25.61	16.81	13.79	35.20	23.58	20.08	–	–	–	–	0.10	1 core @ 2.5 GHz (Python)	×
CMKD [161]	2022	95.14	90.28	83.91	28.55	18.69	16.77	38.98	25.82	22.80	–	–	–	–	0.10	1 core @ 2.5 GHz (C/C++)	✓
MonoPGC [162]	2023	–	–	–	24.68	17.17	14.14	32.50	23.14	20.30	–	–	–	–	–	–	×
MonoATT [163]	2023	–	–	–	24.72	17.37	15.00	36.87	24.42	21.88	–	–	–	–	–	–	×
NeurOCS [164]	2023	96.39	91.08	81.20	29.89	18.94	15.90	37.27	24.49	20.89	–	–	–	–	0.10	GPU @ 2.5 GHz (Python)	×
MonoNerd [52]	2023	94.60	86.89	77.23	22.75	17.13	15.63	31.13	23.46	20.97	–	–	–	–	NA	1 core @ 2.5 GHz (Python)	✓
MonoSKD [51]	2023	96.68	91.34	83.69	28.43	17.35	15.01	37.12	24.08	20.37	–	–	–	–	0.04	1 core @ 2.5 GHz (Python)	✓
ODM3D [165]	2023	–	–	–	29.75	19.09	16.93	39.41	26.02	22.76	–	–	–	–	–	–	✓
MonoUNI [166]	2023	94.30	88.96	78.95	24.75	16.73	13.49	33.28	23.05	19.39	–	–	–	–	0.04	1 core @ 2.5 GHz (Python)	×
MonoDSSM [167]	2024	93.96	88.31	76.15	21.47	14.55	11.78	28.29	19.59	16.34	–	–	–	–	0.02	1 core @ 2.5 GHz (Python + C/C++)	×
MonoCD [53]	2024	96.43	92.91	85.55	25.53	16.59	14.53	33.41	22.81	19.57	–	–	–	–	NA	1 core @ 2.5 GHz (Python)	✓
MonoMAE [168]	2024	–	–	–	25.60	18.84	16.78	34.15	24.93	21.76	–	–	–	–	–	–	×
MonoDiff [169]	2024	–	–	–	30.18	21.02	18.16	–	–	–	–	–	–	–	–	–	×
MonoDFNet [170]	2024	–	–	–	25.71	19.07	15.96	33.56	24.52	21.09	–	–	–	–	–	–	✓
DPL [171]	2024	–	–	–	24.19	16.67	13.83	33.16	22.12	18.74	–	–	–	–	–	–	×
Dp-M3D [172]	2025	–	–	–	23.41	13.65	12.91	32.38	20.13	16.58	–	–	–	–	–	–	×
MonoDINO-DETR [173]	2025	–	–	–	–	–	–	–	–	–	–	–	–	–	–	–	✓
Pseudo-LiDAR2D [174]	2025	–	–	–	–	–	–	–	–	–	–	–	–	–	–	–	×
***Stereo Camera:***																	
3DOP [175]	2015	92.96	89.55	79.38	–	–	–	–	–	–	–	–	–	–	3.00	GPU @ 2.5 GHz (Matlab + C/C++)	×
Pseudo-LiDAR [176]	2018	85.40	67.79	58.50	54.53	34.05	28.25	67.30	45.00	38.40	–	–	–	–	0.40	GPU @ 2.5 GHz (Python + C/C++)	✓
Stereo R-CNN [54]	2019	93.98	85.98	71.25	47.58	30.23	23.72	61.92	41.31	33.42	–	–	–	–	0.30	GPU @ 2.5 GHz (Python)	✓
TLNet [177]	2019	76.92	63.53	54.58	7.64	4.37	3.74	13.71	7.69	6.73	–	–	–	–	0.10	1 core @ 2.5 GHz (Python)	✓
Pseudo-LiDAR++ [178]	2019	94.46	82.90	75.45	61.11	42.43	36.99	78.31	58.01	51.25	–	–	–	–	0.40	GPU @ 2.5 GHz (Python)	✓
RT3D-Stereo [179]	2019	56.53	45.81	37.63	29.90	23.28	18.96	58.81	46.82	38.38	–	–	–	–	0.08	GPU @ 2.5 GHz (C/C++)	✓
DSGN [55]	2020	95.53	86.43	78.75	73.50	52.18	45.14	82.90	65.05	56.60	–	–	–	–	0.67	NVIDIA Tesla V100	✓
OC-Stereo [180]	2020	87.39	74.60	62.56	55.15	37.60	30.25	68.89	51.47	42.97	–	–	–	–	0.35	1 core @ 2.5 GHz (Python + C/C++)	✓
ZoomNet [181]	2020	94.22	83.92	69.00	55.98	38.64	30.97	72.94	54.91	44.14	–	–	–	–	0.30	1 core @ 2.5 GHz (C/C++)	✓
Disp R-CNN [182]	2020	93.45	82.64	70.45	68.21	45.78	37.73	79.76	58.62	47.73	–	–	–	–	0.387	GPU @ 2.5 GHz (Python + C/C++)	✓
Pseudo-LiDAR E2E [183]	2020	–	–	–	64.75	43.92	38.14	79.60	58.80	52.10	–	–	–	–	–	–	✓
CDN [184]	2020	95.85	87.19	79.43	74.52	54.22	46.36	83.32	66.24	57.65	–	–	–	–	0.60	GPU @ 2.5 GHz (Python)	✓
CG-Stereo [185]	2020	96.31	90.38	82.80	74.39	53.58	46.50	85.29	66.44	58.95	–	–	–	–	0.57	GeForce RTX 2080 Ti	×
RTS3D [186]	2020	–	–	–	58.51	37.38	31.12	72.17	45.22	38.48	–	–	–	–	–	–	✓
RT3D-GMP [187]	2020	62.41	51.95	39.14	16.23	11.41	10.12	69.14	59.00	45.49	–	–	–	–	0.06	GPU @ 2.5 GHz (Python + C/C++)	×
YOLOStereo3D [56]	2021	94.81	82.15	62.17	65.68	41.25	30.42	76.10	50.28	36.86	–	–	–	–	0.10	GPU 1080Ti	✓
SIDE [188]	2021	–	–	–	47.69	30.82	25.68	–	–	–	–	–	–	–	–	–	×
LIGA-Stereo [57]	2021	96.43	93.82	86.19	81.39	64.66	57.22	88.15	76.78	67.40	–	–	–	–	0.40	1 core @ 2.5 GHz (Python + C/C++)	✓
StereoCenterNet [189]	2021	96.61	91.27	93.50	49.44	31.30	25.62	62.97	42.12	35.37	–	–	–	–	0.04	GPU @ 2.5 GHz (Python)	×
ESGN [190]	2021	44.09	32.60	29.10	65.80	46.39	38.42	78.10	58.12	49.28	–	–	–	–	0.06	GPU @ 2.5 GHz (Python + C/C++)	×
Pseudo-Stereo [191]	2022	95.75	90.27	82.32	23.74	17.74	15.14	32.64	23.76	20.64	–	–	–	–	0.25	1 core @ 2.5 GHz (C/C++)	✓
DSGN++ [192]	2022	98.08	95.70	88.27	83.21	67.37	59.91	88.55	78.94	69.74	–	–	–	–	0.20	GeForce RTX 2080 Ti	✓
DID-M3D [193]	2022	94.29	91.04	81.31	24.40	16.29	13.75	32.95	22.76	19.83	–	–	–	–	0.04	1 core @ 2.5 GHz (Python)	✓
DMF [194]	2022	89.50	85.49	82.52	77.55	67.33	62.44	84.64	80.29	76.05	–	–	–	–	0.20	1 core @ 2.5 GHz (Python + C/C++)	×
StereoDistill [58]	2023	97.61	93.43	87.71	81.66	66.39	57.39	89.03	78.59	69.34	–	–	–	–	0.40	1 core @ 2.5 GHz (Python)	×
PS-SVDM [195]	2023	94.49	87.55	78.21	29.22	18.13	15.35	38.18	24.82	20.89	–	–	–	–	1.00	1 core @ 2.5 GHz (Python)	×
***Multi-View Camera:***																	
3DOMV [96]	2017	–	–	–	–	–	–	–	–	–	–	–	–	–	–	–	×
MVRA [196]	2019	95.87	94.98	82.52	5.19	3.27	2.49	9.05	5.84	4.50	–	–	–	–	0.18	GPU @ 2.5 GHz (Python)	×
DETR3D [59]	2021	–	–	–	–	–	–	–	–	–	41.2	47.9	–	–	–	–	×
BEVDet [197]	2021	–	–	–	–	–	–	–	–	–	39.8	46.3	–	–	–	–	×
BEVDepth [198]	2022	–	–	–	–	–	–	–	–	–	52	60.9	–	–	–	–	×

**Table 4:** LiDAR-based 3D object detection results on KITTI car test set, nuScenes test set, and Waymo validation set.

Method	Year	AP_2D_			AP_3D_			AP_BEV_			nuScenes		Waymo		Time (s)	Hardware	Code Available
		E	M	H	E	M	H	E	M	H	mAP	NDS	L1 mAP	L2 mAP			
ImVoxelNet [199]	2022	89.90	79.09	69.45	17.15	10.97	9.15	25.19	16.37	13.58	41.2	47.9	–	–	0.20	GPU @ 2.5 GHz (Python)	✓
PETR [60]	2022	–	–	–	–	–	–	–	–	–	44.5	50.4	–	–	–	–	×
STS [200]	2022	–	–	–	–	–	–	–	–	–	42.2	52.9	–	–	–	–	×
BEVerse [201]	2022	–	–	–	–	–	–	–	–	–	39.3	53.1	–	–	–	–	×
BEVFormer [61]	2022	–	–	–	–	–	–	–	–	–	48.1	56.9	–	–	–	–	×
SOLOFusion	2022	–	–	–	–	–	–	–	–	–	54.0	61.9	–	–	–	–	×
PolarFormer [202]	2022	–	–	–	–	–	–	–	–	–	45.6	54.3	–	–	–	–	×
FocalPETR [203]	2022	–	–	–	–	–	–	–	–	–	46.5	51.6	–	–	–	–	×
BEV Distill [204]	2022	–	–	–	–	–	–	–	–	–	49.6	59.4	–	–	–	–	×
HoP [205]	2023	–	–	–	–	–	–	–	–	–	62.4	68.5	–	–	–	–	×
SparseBEV [62]	2023	–	–	–	–	–	–	–	–	–	60.3	67.5	–	–	–	–	×
StreamPETR [206]	2023	–	–	–	–	–	–	–	–	–	55.0	63.1	–	–	–	–	×
PolarBEVDet [207]	2024	–	–	–	–	–	–	–	–	–	55.8	63.5	–	–	–	–	×
RoPETR [63]	2025	–	–	–	–	–	–	–	–	–	64.8	70.9	–	–	–	–	×
***Projection-Based:***																	
C-YOLO [68]	2018	–	–	–	67.72	64.00	63.01	85.89	77.40	77.33	–	–	–	–	–	–	✓
TopNet [208]	2018	58.04	45.85	41.11	12.67	9.28	7.95	80.16	68.16	63.43	–	–	–	–	0.01	NVIDIA GeForce 1080 Ti (TF-GPU)	×
BirdNet [69]	2018	79.30	57.12	55.16	40.99	27.26	25.32	84.17	59.83	57.35	–	–	–	–	0.11	Titan Xp (Caffe)	✓
PIXOR [67]	2019	–	–	–	–	–	–	81.70	77.05	72.95	–	–	–	–	–	–	✓
FVNet [209]	2019	86.14	77.19	69.27	65.43	57.34	51.85	78.04	65.03	57.89	–	–	–	–	–	–	✓
MODet [210]	2019	66.06	62.54	60.04	–	–	–	90.80	87.56	82.69	–	–	–	–	0.05	GTX1080Ti	×
HDNet [211]	2020	–	–	–	–	–	–	89.14	86.57	78.32	–	–	–	–	–	–	✓
PIXOR++ [211]	2020	–	–	–	–	–	–	93.28	86.01	80.11	–	–	–	–	–	–	×
BirdNet+ [212]	2021	92.61	86.73	81.80	76.15	64.04	59.79	87.43	81.85	75.36	–	–	–	–	0.11	Titan Xp (Caffe)	✓
MGTANet [213]	2022	–	–	–	–	–	–	–	–	–	67.50	72.70	–	–	–	–	✓
GPA3D [214]	2023	–	–	–	–	–	–	–	–	–	–	–	–	–	–	–	✓
***Voxel-Based:***																	
Vote3D [215]	2015	–	–	–	–	–	–	–	–	–	–	–	–	–	–	–	×
VoxelNet [75]	2017	–	–	–	77.82	64.17	57.51	87.95	78.39	71.29	–	–	–	–	–	–	×
SECOND [76]	2018	–	–	–	83.13	73.66	66.20	89.39	83.77	78.59	–	–	–	–	–	–	✓
PointPillars [77]	2018	94.00	91.19	88.17	82.58	74.31	68.99	90.07	86.56	82.81	40.10	55.00	–	–	0.016	1080 Ti + Intel i7	✓
HotSpotNet [78]	2019	96.21	92.81	89.80	87.60	78.31	73.34	94.06	88.09	83.24	59.30	66.00	–	–	0.04	1 core @ 2.5 GHz (Py + C/C++)	×
Voxel R-CNN [79]	2020	96.49	95.11	92.45	90.90	81.62	77.06	94.85	88.83	86.13	–	–	75.59	66.59	0.04	GPU @ 3.0 GHz (C/C++)	✓
VoTr-TSD [80]	2021	95.95	94.81	92.24	89.90	82.09	79.14	94.03	90.34	86.14	–	–	74.95	65.91	0.07	1 core @ 2.5 GHz (C/C++)	✓
TED [81]	2022	96.64	96.03	93.35	91.61	85.28	80.68	95.44	92.05	87.30	–	–	–	–	0.10	1 core @ 2.5 GHz (C/C++)	✓
VoxSeT [216]	2022	96.16	95.23	90.49	88.53	82.06	77.46	92.70	89.07	86.29	–	–	–	–	0.033	1 core @ 2.5 GHz (C/C++)	✓
FocalsConv [217]	2022	96.30	95.28	92.69	90.55	82.28	77.59	92.67	89.00	86.33	–	–	–	–	0.10	1 core @ 2.5 GHz (C/C++)	✓
PillarNet [218]	2022	–	–	–	–	–	–	–	–	–	66.00	71.40	83.23	76.09	–	–	✓
SWFormer [219]	2022	–	–	–	–	–	–	–	–	–	–	–	77.8	69.2	–	–	×
PV-GNN [220]	2024	–	–	–	91.64	82.49	77.28	95.09	92.38	87.44	–	–	–	–	–	–	×
***Point-Based:***																	
iPOD [221]	2018	90.20	89.30	87.37	71.40	53.46	48.34	86.93	83.98	77.85	–	–	–	–	–	–	×
PointRCNN [72]	2018	95.92	91.90	87.11	86.96	75.64	70.70	92.13	87.39	82.72	–	–	–	–	0.10	GPU @ 2.5 GHz (Py + C/C++)	✓
STD [222]	2019	96.14	93.22	90.53	87.95	79.71	75.09	94.74	89.19	86.42	–	–	–	–	0.08	GPU @ 2.5 GHz (Py + C/C++)	✓
PointRGCN [223]	2019	96.19	92.67	87.66	85.97	75.73	70.60	91.63	87.49	90.73	–	–	–	–	0.26	GPU @ V100 (Python)	✓
3DSSD [224]	2020	97.69	95.10	92.18	88.36	79.57	74.55	92.66	89.02	85.86	42.60	56.40	–	–	0.04	GPU @ 2.5 GHz (Py + C/C++)	✓
Point-GNN [225]	2020	96.58	93.50	88.35	88.33	79.47	72.29	93.11	89.17	83.90	–	–	–	–	0.60	GPU @ 2.5 GHz (Python)	✓
PointFormer [74]	2020	–	–	–	87.13	77.06	69.25	–	–	–	53.60	–	–	–	–	–	✓
EPNet++ [226]	2021	96.73	95.17	92.10	91.37	81.96	76.71	95.41	89.00	85.73	–	–	–	–	0.10	GPU @ 2.5 GHz (Python)	✓
SASA [227]	2022	96.01	95.35	92.42	88.76	82.16	77.16	92.87	89.51	86.35	–	–	–	–	0.04	1 core @ 2.5 GHz (Py + C/C++)	✓
IA-SSD [73]	2022	96.10	93.56	90.68	88.27	80.32	75.10	92.79	89.33	84.35	–	–	–	–	0.014	1 core @ 2.5 GHz (C/C++)	✓
DFAF3D [228]	2023	96.58	93.32	90.24	88.59	79.37	72.21	93.14	89.45	84.22	–	–	–	–	–	1 core @ 2.5 GHz (Python)	×
HINTED [229]	2024	95.16	90.97	85.55	84.00	74.13	67.03	90.61	86.01	79.29	–	–	–	–	0.04	1 core @ 2.5 GHz (C/C++)	✓
***Point–Voxel Hybrid:***																	
PVCNN [230]	2019	–	–	–	–	–	–	–	–	–	–	–	–	–	–	–	✓
Fast Point R-CNN [82]	2019	96.13	93.18	87.68	85.29	77.40	70.24	90.87	87.84	80.52	–	–	–	–	0.06	GPU @ 2.5 GHz (Py + C/C++)	×
PV-RCNN [83]	2019	98.17	94.70	92.04	90.25	81.43	76.82	94.98	90.65	86.14	–	77.51	–	–	0.08	1 core @ 2.5 GHz (Py + C/C++)	✓
SA-SSD [231]	2020	97.92	95.16	90.15	88.75	79.79	74.16	95.03	91.03	85.96	–	–	–	–	0.04	1 core @ 2.5 GHz (Python)	✓
BADet [232]	2021	98.65	95.34	90.28	89.28	81.61	76.59	95.23	91.32	86.48	–	–	–	–	0.14	1 core @ 2.5 GHz (C/C++)	✓
Pyramid-PV [233]	2021	95.88	95.13	92.62	88.39	82.08	77.49	92.19	88.84	86.21	–	–	–	–	0.07	1 core @ 2.5 GHz (C/C++)	✓
DVFENet [234]	2021	95.35	94.57	91.77	86.20	79.18	74.58	90.93	87.68	84.60	–	–	–	–	0.05	1 core @ 2.5 GHz (Py + C/C++)	×
PDV [84]	2022	96.07	95.00	92.44	90.43	81.86	77.36	94.56	90.48	86.23	–	–	–	–	0.10	1 core @ 2.5 GHz (C/C++)	✓
EQ-PVRCNN [235]	2022	98.23	95.32	92.65	90.13	82.01	77.53	94.55	89.09	86.40	–	–	–	–	0.20	GPU @ 2.5 GHz (Py + C/C++)	✓
PVT-SSD [236]	2023	96.75	95.90	90.69	90.65	82.29	76.85	95.23	91.63	86.43	–	–	–	–	0.05	1 core @ 2.5 GHz (Py + C/C++)	×
PG-RCNN [237]	2023	96.66	95.40	90.55	89.38	82.13	77.33	93.39	89.46	86.54	–	–	–	–	0.06	GPU @ 1.5 GHz (Python)	✓
Uni3DETR [238]	2023	–	–	–	91.14	82.26	77.58	–	–	–	–	–	–	–	–	–	✓

**Table 5:** Radar-based 3D object detection results on KITTI car test set, nuScenes test set, and Waymo validation set.

Method	Year	AP_2D_			AP_3D_			AP_BEV_			nuScenes		Waymo		Time (s)	Hardware	Code Available
		E	M	H	E	M	H	E	M	H	mAP	NDS	L1	L2			
Radar-PointGNN [86]	2021	–	–	–	–	–	–	–	–	-	0.5	3.4	–	–	–	–	×
K-Radar [87]	2022	–	–	–	–	–	–	–	–	–	–	–	–	–	–	–	✓
KPConvPillars [88]	2022	–	–	–	–	–	–	–	–	–	4.9	13.9	–	–	–	–	×
Dual Radar [239]	2023	–	–	–	–	–	–	–	–	–	–	–	–	–	–	–	×
CenterRadarNet [240]	2024	–	–	–	–	–	–	–	–	–	–	–	–	–	–	–	×
RadarDistill [89]	2024	–	–	–	–	–	–	–	–	-	20.5	43.7	–	–	–	–	✓
RADLER [90]	2025	–	–	–	–	–	–	–	–	–	–	–	–	–	–	–	✓

**Table 6:** Multi-modal-based 3D object detection results on KITTI car test set, nuScenes test set, and Waymo validation set.

Method	Year	AP_2D_			AP_3D_			AP_BEV_			nuScenes		Waymo		Time (s)	Hardware	Code Available
		E	M	H	E	M	H	E	M	H	mAP	NDS	L1	L2			
***Early Fusion:***																	
F-PointNet [92]	2017	95.85	95.17	85.42	82.19	69.79	60.59	91.17	84.67	74.77	–	–	–	–	0.17	GPU @ 3.0 GHz (Python)	✓
F-ConvNet [93]	2019	95.85	92.19	80.09	87.36	76.39	66.69	91.51	85.84	76.11	–	–	–	–	0.47	GPU @ 2.5 GHz (Python + C/C++)	✓
RoarNet [241]	2018	–	–	–	–	–	–	–	–	–	–	–	–	–	–	–	×
Complexer-YOLO [242]	2019	91.92	84.16	79.62	55.93	47.34	42.60	77.24	68.96	64.95	–	–	–	–	0.06	GPU @ 3.5 GHz (C/C++)	✓
PointPainting [91]	2019	98.39	92.58	89.71	82.11	71.70	67.08	92.45	88.11	83.36	–	–	–	–	0.40	GPU @ 2.5 GHz (Python + C/C++)	×
FusionPainting [243]	2021	–	–	–	–	–	–	–	–	–	–	–	–	–	–	–	×
MVP [244]	2021	–	–	–	–	–	–	–	–	–	–	–	–	–	–	–	×
F-PointPillars [94]	2021	–	–	–	–	–	–	–	–	–	–	–	–	–	0.06	4 cores @ 3.0 GHz (Python)	✓
PointAugmenting [245]	2021	–	–	–	–	–	–	89.14	86.57	78.32	–	–	–	–	–	–	✓
VirConvNet [95]	2023	98.00	97.27	94.53	92.48	87.20	82.45	95.99	93.52	90.38	–	–	–	–	0.09	1 core @ 2.5 GHz (C/C++)	✓
HDF [246]	2025	–	–	–	–	–	–	–	–	–	–	–	–	–	–	–	×
***Mid-Level Fusion:***																	
MV3D [96]	2016	96.47	90.83	78.63	74.97	63.63	54.00	86.62	78.93	69.80	–	–	–	–	0.36	GPU @ 2.5 GHz (Python + C/C++)	✓
AVOD [97]	2017	95.17	89.88	82.83	76.39	66.47	60.23	89.75	84.95	78.32	–	–	–	–	0.08	Titan X (Pascal)	✓
PointFusion [247]	2017	–	–	–	–	–	–	–	–	–	–	–	–	–	–	–	×
ContFuse [98]	2018	–	–	–	83.68	68.78	61.67	94.07	85.35	75.88	–	–	–	–	0.06	GPU @ 2.5 GHz (Python)	×
MVXNet [248]	2019	–	–	–	83.20	72.70	65.20	–	–	–	–	–	–	–	–	–	×
PI-RCNN [249]	2019	96.17	92.66	87.68	84.37	74.82	70.03	91.44	85.81	81.00	–	–	–	–	0.10	1 core @ 2.5 GHz (Python)	×
MCF3D [250]	2019	–	–	–	–	–	–	–	–	–	–	–	–	–	–	–	×
MMF [99]	2020	97.41	94.25	91.80	88.40	77.43	70.22	93.67	88.21	81.99	–	–	–	–	0.08	GPU @ 2.5 GHz (Python)	×
3D-CVF [251]	2020	96.78	93.36	86.11	89.20	80.05	73.11	93.52	89.56	82.45	–	–	–	–	0.06	1 core @ 2.5 GHz (C/C++)	✓
EPNet [100]	2020	96.15	94.44	89.99	89.81	79.28	74.59	94.22	88.47	83.69	–	–	–	–	0.10	1 core @ 2.5 GHz (Python + C/C++)	✓
EPNet++ [226]	2021	96.73	95.17	92.10	91.37	81.96	76.71	95.41	89.00	85.73	–	–	–	–	0.10	GPU @ 2.5 GHz (Python)	×
TransFusion [101]	2022	–	–	–	–	–	–	–	–	–	68.90	71.70	–	–	–	–	×
BEVFusion [252]	2022	–	–	–	–	–	–	–	–	–	–	–	–	–	–	–	×
FUTR3D [102]	2022	–	–	–	–	–	–	–	–	–	69.40	72.10	–	–	–	–	×
DeepFusion [253]	2022	–	–	–	–	–	–	–	–	–	–	–	–	–	–	–	×
MSMDFusion [254]	2022	–	–	–	–	–	–	–	–	–	–	–	–	–	–	–	×
CAT-Det [255]	2022	95.97	94.71	92.07	89.87	81.32	76.68	92.59	90.07	85.82	–	–	–	–	0.30	GPU @ 2.5 GHz (Python + C/C++)	×
HMFI [256]	2022	96.29	95.16	92.45	88.90	81.93	77.30	93.04	89.17	86.37	–	–	–	–	0.10	1 core @ 2.5 GHz (C/C++)	✓
LoGoNet [257]	2023	96.60	95.55	93.07	91.80	85.06	80.74	95.48	91.52	87.09	–	–	–	–	0.10	1 core @ 2.5 GHz (C/C++)	✓
SDVRF [258]	2023	–	–	–	–	–	–	–	–	–	–	–	–	–	–	–	×
SupFusion [259]	2023	–	–	–	–	–	–	–	–	–	–	–	–	–	–	–	×
FGFusion [260]	2023	–	–	–	–	–	–	–	–	–	–	–	–	–	–	–	×
VCD [261]	2023	–	–	–	–	–	–	–	–	–	–	–	–	–	–	–	×
UniTR [262]	2023	–	–	–	–	–	–	–	–	–	70.90	74.50	–	–	–	–	×
***Late Fusion:***																	
CLOCS [103]	2020	96.77	96.07	91.11	89.16	82.28	77.23	92.91	89.48	86.42	–	–	–	–	0.10	1 core @ 2.5 GHz (Python)	×
Fast-CLOCS [104]	2022	96.69	95.75	90.95	89.10	80.35	76.99	93.03	89.49	86.40	63.10	68.70	–	–	0.10	GPU @ 2.5 GHz (Python)	✓

## 5. Evaluation and Discussion

Camera-based methods are lightweight, cost-effective, and easy to deploy, making them particularly attractive for applications with limited hardware constraints. They preserve rich semantic cues such as texture, colour, and object appearance, which are advantageous for visual understanding and object classification. However, due to the inherent challenge of regressing depth from 2D projections, camera-based detectors often underperform in spatial metrics such as 3D IoU, localization precision, and orientation accuracy [29].

Stereo and multi-view systems partially mitigate these issues by leveraging geometric constraints, enabling disparity estimation and improved depth perception. Despite this, their accuracy still lags behind that of LiDAR-based systems, especially in large-scale outdoor environments. Multi-camera configurations, while offering broader spatial coverage, require complex calibration and are mostly reported on datasets such as nuScenes, which support full 360° camera rings.

LiDAR sensors, by contrast, directly capture 3D spatial coordinates, providing accurate and dense measurements of the environment. This makes them particularly effective for estimating object position, size, and orientation. LiDAR-based methods consistently outperform camera-only approaches in 3D detection tasks and show greater robustness under varying lighting conditions [24]. However, they come with their own drawbacks: LiDAR hardware is significantly more expensive, bulkier, and prone to performance degradation in adverse weather (e.g., fog, rain, or snow) [32]. Additionally, point density decreases with distance, reducing resolution for far-away objects. Radars solve some of these problems but still lack the accuracy and resolution of LiDAR.

To overcome the individual limitations of single-sensor systems, multi-modal fusion has become an increasingly prevalent approach. By integrating LiDAR’s spatial precision with the semantic richness of images, fusion-based models yield improved scene understanding, higher detection accuracy, and better robustness to occlusions or sensor dropout [29]. Nonetheless, these methods introduce new complexities: precise extrinsic calibration is required, and data from different sensors must be carefully synchronized and spatially aligned. Fusion also increases computational burden, making real-time inference more challenging.

Table 3, Table 4, Table 5 and Table 6 provide a comprehensive evaluation of 3D OD performance across a wide range of methods, organized by sensor modality, representation type, and publication year. Each table reports accuracy metrics on the KITTI, nuScenes, and Waymo datasets, with additional inference time metrics (reported for KITTI) and public code availability. This taxonomy enables direct comparisons between monocular, stereo, LiDAR-only, radar-only, and fusion-based pipelines.

A consistent hierarchy emerges: LiDAR-based methods outperform monocular and stereo-based ones in 3D localization, while multi-modal methods tend to achieve the best overall results. These trends underscore the inherent difficulty of monocular depth estimation and highlight the advantages of incorporating complementary sensory cues.

From early 2D-proposal-based monocular models to state-of-the-art transformer-based fusion frameworks, the field of 3D OD has progressively shifted toward hybrid pipelines that balance spatial accuracy, semantic reasoning, and computational efficiency. This evolution is reflected not only in architectural choices but also in quantitative improvements on benchmark datasets.

An important trend highlighted in the tables is the trade-off between inference speed and detection accuracy. While transformer-based and two-stage architectures dominate in terms of AP and 3D IoU scores, single-stage detectors and projection-based models have gained traction for real-time deployment. For instance, models such as PointPillars, PIXOR, and SMOKE offer reduced latency while maintaining competitive accuracy. Fusion-based methods like TransFusion and EPNet achieve high accuracy but are computationally heavier, whereas late fusion methods like Fast-CLOCs enable faster inference with minimal architectural change.

Recent models such as TED, PV-RCNN, and CenterFusion exemplify the growing ability to combine high accuracy with low latency, even in multi-modal setups. TED currently holds state-of-the-art performance among LiDAR-only methods on the KITTI benchmark, while PV-RCNN combines voxel-grid structure with point-level refinement for balanced performance. BEV and pillar-based projections dominate low-latency deployment scenarios, especially in AD settings where speed is critical.

Focusing specifically on camera-based methods, steady gains in AP have been observed over time. Monocular detectors, though inherently limited by depth ambiguity, have benefited from advances in deep regression, geometric priors, and knowledge distillation. Stereo and multi-view approaches improve upon monocular baselines by leveraging depth cues, with many of the highest-performing models being reported on the nuScenes benchmark due to its extensive camera coverage. Inference times have also improved over time, making real-time deployment increasingly feasible.

On the other hand, radar-only based methods still showcase limited accuracy performance in the few reported metrics on benchmarked datasets. They lack the resolution and semantic information capturing required for proper 3D understanding. However, they can provide information that other sensors cannot and can operate in more extreme environments where other sensors are less suitable, thereby greatly reinforcing a system’s robustness and reliability when fused. Still, works like [263] highlight how radar-only 3D object detection methods can achieve success in specific applications and how this area holds significant potential for further advancement.

In summary, there is no universally optimal modality or representation for 3D OD. Each approach entails trade-offs between semantic richness, geometric fidelity, latency, cost, and robustness. The ideal method depends on application-specific constraints such as sensor availability, environmental variability, and real-time requirements. Future research will likely focus on three main directions: improving robustness to sensor degradation, optimizing fusion architectures for scalable deployment, and developing more generalizable representations that support cross-domain transfer.

As foundation models and self-supervised learning continue to mature, they will likely play a central role in the next generation of 3D OD systems. Scalable, modality-agnostic perception frameworks that unify sparse and dense data sources will be critical in enabling safe and reliable autonomous operation across diverse scenarios.

Table 3, Table 4, Table 5 and Table 6 constitute the core of this survey. Together, they provide a structured and comprehensive summary of the most influential 3D OD methods developed over the past decade. A total of 205 methods were reviewed and are reported in these tables, spanning a wide range of input modalities, data representations, and architectural paradigms. These methods are categorized by sensor type (monocular, stereo, multi-camera, LiDAR, and fusion-based), ordered chronologically by publication year, and benchmarked using standardized metrics from the KITTI, nuScenes, and Waymo datasets. Inference time (when available) is also included for the KITTI benchmark, along with an indicator of public code availability.

This structured compilation enables readers to trace the chronological and methodological evolution of the field, compare performance trends across modalities, and identify prevailing design trade-offs. It also serves as a reference point for researchers seeking to position new contributions within the broader landscape.

To facilitate wider access and enable dynamic exploration of 3D OD methods, a dedicated online repository was created: (https://3d-object-detection-hub.github.io/, accessed on 25 April 2025). This interactive website contains an extended collection of detection models, grouped by input modality, data representation, and publication year. The platform is designed to be continuously updated and searchable, supporting users in exploring methods across monocular-, stereo-, LiDAR-, radar-, and fusion-based pipelines.

The construction of this survey involved a multi-stage methodology. Initially, existing review papers and survey studies were consulted to establish a foundational taxonomy of input modalities, processing strategies, and detection paradigms. These sources provided both historical context and the initial references for influential models. From there, models were categorized according to the taxonomy developed in Section 4, distinguishing between representation types such as point-based, voxel-based, projection-based and hybrid models.

To obtain reliable performance metrics, official benchmark leaderboards, such as those of KITTI, nuScenes, and Waymo, were systematically reviewed. For each method, metrics were gathered from the benchmark websites whenever publicly available. When benchmark entries were missing or incomplete, values were extracted directly from the original publications. Inference time was also recorded, with particular emphasis on KITTI runtime to compare real-time capability. All data presented in Table 3, Table 4, Table 5 and Table 6 were manually verified to ensure consistency, dataset compatibility, and fair metric alignment across methods. Where necessary, metadata were aggregated from supplementary material or code repositories to fill gaps in the original publications.

Each table entry is annotated by sensor modality (e.g., monocular, stereo, LiDAR, multi-modal) and representation type (e.g., voxel, point, projection, hybrid). Methods are listed chronologically by year of publication to illustrate architectural progression. Accuracy metrics are reported separately for the KITTI, nuScenes, and Waymo datasets, providing cross-benchmark comparability. An additional column denotes whether the method’s codebase is publicly available, enabling practical reproducibility and further experimentation.

This work thus aims to offer one of the most comprehensive and up-to-date surveys of the 3D OD methods available. In contrast to prior surveys that focus narrowly on single-modality pipelines, this review takes a modality-agnostic perspective and includes both classical and SoA methods up to May 2025, capturing recent advances that have not yet appeared in other reviews or benchmark summary papers.

## 6. Conclusions

This work presented a comprehensive survey of 3D object detection methods, spanning monocular, stereo, multi-camera, radar, LiDAR, and multi-modal fusion approaches. A consistent taxonomy was proposed to organize the field, covering different input modalities and data representations. The survey traced the historical development of these methods and benchmarked them using metrics from publicly available datasets such as KITTI, nuScenes, and Waymo. In total, 205 methods were analysed, and their performance was synthesized across accuracy, inference time, and implementation availability.

Monocular methods are the most lightweight and scalable but are limited by their lack of direct depth sensing. Stereo systems partially address this with geometric priors, though their effectiveness declines at longer distances and under occlusion. LiDAR-based methods excel in spatial accuracy and robustness but are hindered by high cost, sparse point clouds at long ranges, and degraded performance in adverse weather. Radar-based methods exist and can be a good choice for specific use cases but are usually coupled with other sensors. Multi-modal fusion strategies consistently achieve superior accuracy and resilience by combining complementary sensor inputs, but they also introduce additional complexity in calibration, synchronization, and training.

Beyond benchmarking, the survey consolidates fragmented knowledge from recent papers, official leaderboards, and existing reviews into a unified and accessible framework. The dedicated website accompanying this thesis serves as a continuously updated repository of methods and results, supporting reproducibility and further research. Overall, this work contributes a modality-agnostic, data-driven, and taxonomically structured reference for 3D perception in autonomous systems.

The field of 3D object detection continues to evolve rapidly, with new architectural paradigms and sensing modalities expanding its capabilities. Recent trends show a gradual transition from purely CNN-based architectures to Transformer-based models that better capture long-range dependencies and global context. Other emerging paradigms such as knowledge distillation, NeRF-based scene representations, and foundation models trained on large-scale multimodal data are also gaining traction. The field is moving toward scalable, robust real-time solutions suitable for deployment in real-world AV systems. Heavy focus is being placed on enhancing robustness under challenging conditions and ensuring scalability for large-scale AV deployments. Techniques that reduce latency, energy consumption, and hardware requirements, without sacrificing detection accuracy, will be key enablers of future perception systems. The groundwork laid by this work provides a strong foundation for further exploration into robust, efficient, and generalizable 3D detection pipelines for autonomous applications.

## Figures and Tables

**Table 1:** Exteroceptive sensors performance comparison.

Sensor	Range	Accuracy	Cost	Comput. Cost	Size	Depth	Colour	Affected by Illumination	Affected by Weather
Monocular Camera	Medium	Medium	Low	High	Small	No	Yes	Yes	Yes
Stereo Camera	Medium	Medium	Medium	High	Medium	Yes	Yes	Yes	Yes
Infrared Camera	Medium	Medium	Low	Medium	Small	No	No	No	Yes
Sonar/Ultrasonic	Low	Low	Low	Low	Small	Yes	No	No	No
Radar	High	Medium	Medium	Medium	Medium	Yes	No	No	No
LiDAR	High	High	High	Medium	Large	Yes	No	No	Yes

**Table 2:** Common datasets for 3D object detection in autonomous vehicles.

Dataset	Year	# Cameras	# LiDARs	# Scenes	# Classes	Locations	Night	Rain	Annotated 3D BBoxes	Annotated Frames
KITTI [28]	2012	2	1	22	3	Germany	No	No	80k	15k
ApolloScape [38]	2018	2	2	73	27	China	Yes	No	70k	80k
nuScenes [36]	2019	6	1	1000	23	USA/Singapore	Yes	Yes	1.4M	40k
ArgoVerse [39]	2019	9	2	113	15	USA	Yes	Yes	993K	22k
Waymo Open [37]	2019	5	5	1150	4	USA	Yes	Yes	12M	230k
Lyft Level 5 [40]	2019	7	3	366	9	USA	No	No	1.3M	46k
H3D [41]	2019	3	1	160	8	USA	No	No	1.1M	27k

## Data Availability

The original data presented in the study will be made openly available at GitHub upon publication.

## Abbreviations

The following abbreviations are used in this manuscript:

AD	Autonomous Driving
ADAS	Advanced Driver Assistance Systems
AI	Artificial Intelligence
AP	Average Precision
AV	Autonomous Vehicle
BEV	Bird’s-Eye View
CNN	Convolutional Neural Network
CV	Computer Vision
DL	Deep Learning
GNSS	Global Navigation Satellite System
IoU	Intersection over Union
IMU	Inertial Measurement Unit
LiDAR	Light Detection and Ranging
mAP	Median Average Precision
ML	Machine Learning
NDS	nuScenes Detection Score
NDS	Non-Maximum Suppression
NN	Neural Network
OD	Object Detection
PC	Point Cloud
Radar	Radio Detection and Ranging
SAE	Society of Automotive Engineers
SoA	State-of-the-Art
Sonar	Sound Navigation and Ranging
SLAM	Simultaneous Localization and Mapping
ToF	Time-of-Flight
